# Acetic Acid-Producing Endophyte *Lysinibacillus fusiformis* Orchestrates Jasmonic Acid Signaling and Contributes to Repression of Cadmium Uptake in Tomato Plants

**DOI:** 10.3389/fpls.2021.670216

**Published:** 2021-06-04

**Authors:** Lin Zhu, Jiansheng Guo, Yujun Sun, Songhua Wang, Cheng Zhou

**Affiliations:** ^1^Key Lab of Bio-Organic Fertilizer Creation, Ministry of Agriculture and Rural Affairs, Anhui Science and Technology University, Bengbu, China; ^2^School of Life Sciences and Technology, Tongji University, Shanghai, China; ^3^School of Medicine, Zhejiang University, Hangzhou, China; ^4^Jiangsu Provincial Key Lab of Solid Organic Waste Utilization, Jiangsu Collaborative Innovation Center of Solid Organic Wastes, Educational Ministry Engineering Center of Resource-Saving Fertilizers, Nanjing Agricultural University, Nanjing, China

**Keywords:** endophytes, jasmonic acid, cadmium toxicity, iron deficiency, acetic acid-producing bacteria

## Abstract

Diverse signaling pathways regulated by phytohormones are essential for the adaptation of plants to adverse environments. Root endophytic bacteria can manipulate hormone-related pathways to benefit their host plants under stress conditions, but the mechanisms underlying endophyte-mediated plant stress adaptation remain poorly discerned. Herein, the acetic acid-producing endophytic bacteria *Lysinibacillus fusiformis* Cr33 greatly reduced cadmium (Cd) accumulation in tomato plants. *L. fusiformis* led to a marked increase in jasmonic acid (JA) content and down-regulation of iron (Fe) uptake-related genes in Cd-exposed roots. Accordantly, acetic acid treatment considerably increased the JA content and inhibited root uptake of Cd uptake. In addition, the Cr33-inoculated roots displayed the increased availability of cell wall and rhizospheric Fe. Inoculation with Cr33 notably reduced the production of nitric oxide (NO) and suppressed Fe uptake systems in the Cd-treated roots, thereby contributing to hampering Cd absorption. Similar results were also observed for Cd-treated tomato plants in the presence of exogenous JA or acetic acid. However, chemical inhibition of JA biosynthesis greatly weakened the endophyte-alleviated Cd toxicity in the plants. Collectively, our findings indicated that the endophytic bacteria *L. fusiformis* effectively prevented Cd uptake in plants via the activation of acetic acid-mediated JA signaling pathways.

## Introduction

Massive industrial waste and the use of phosphate fertilizers cause heavy metal pollution in agricultural soils (Sarwar et al., 2017). Soil cadmium (Cd) is highly mobile and can be transported into the edible tissues of crop plants (Pan et al., 2019). To ensure food safety, sustainable technologies are urgently needed to prevent Cd accumulation in plants grown under Cd-polluted conditions. However, the use of physical and chemical methods to remedy Cd-contaminated soil is impracticable, rendering these methods ineffective for implementation at field sites (Pulford and Watson, 2003). Mounting evidence has indicated that the exploitation of soil-borne bacteria is an emerging alternative for preventing Cd uptake in plants (Ravanbakhsh et al., 2019; Zhou et al., 2019a; Tanwir et al., 2021).

Cd is a toxic metal element for plants that shares chemical similarity with some essential elements such as calcium (Ca), zinc (Zn), and iron (Fe). Cd can compete with other mineral nutrients to bind with several functional proteins, leading to the serious impairment of plant growth (Schützendübel and Polle, 2002). Cd is primarily transported into root cells through some plasma membrane-localized metal transporters such as iron-regulated transporters (IRTs) and zinc/iron-regulated transporter proteins (ZIPs), which results in severe interferences with Fe uptake (Clemens, 2006). Previous studies have indicated that increased Fe supply or rhizospheric Fe availability effectively inhibits Cd uptake in plants, which is closely associated with the competition between Fe and Cd for several metal transporters (Wu et al., 2012; Sebastian and Prasad, 2016). Cd-stressed plants often exhibit typical chlorotic symptoms, similar to those occurring in Fe deficient plants (Chen et al., 2017). Cd stress can trigger Fe deficiency responses, which are accompanied by the up-regulation of Fe acquisition-associated genes including *FIT*, encoding a functional homolog of the bHLH transcription factor Fer, *IRT1* encoding an iron-regulated transporter, and *FRO2*, encoding a putative ferric reduction oxidase (Lešková et al., 2017). Recently, nitric oxide (NO) has been demonstrated to govern Fe deficiency responses in different plant species (Buet et al., 2019). Cd stress can stimulate root NO burst and further initiate signaling pathways resembling those induced by Fe deficiency via the enhancement of *IRT1* and *FRO2* transcripts. Cd-induced *IRT1* expression has also been implicated in the promotion of Cd absorption, thus aggravating Cd toxicity in *Arabidopsis* (Lei et al., 2020). Several phytohormones such as jasmonic acid (JA) and gibberellic acid (GA) negatively regulate the NO-dependent signaling pathways in Cd-stressed plants (Zhu et al., 2012; Lei et al., 2020). In *Arabidopsis*, JA can inhibit root uptake of Cd by reducing root NO accumulation and thus down-regulating *IRT1* expression (Lei et al., 2020). GA has also been reported to repress the root NO burst and the transcription of Cd uptake-related *IRT1*, thereby attenuating Cd toxicity in plants (Zhu et al., 2012). Therefore, the inhibition of NO-mediated *IRT1* expression in Cd-stressed plants is an alternative strategy for interdicting Cd absorption.

Plants coexist with a myriad of soil microbes that play fundamental roles in maintaining plant health and productivity (Cheng et al., 2019). The manipulation of soil-borne bacteria can potentially suppress disease incidence of plants (Liu et al., 2019), increase agricultural production (Gouda et al., 2018), lower the emissions of greenhouse gases (Wu et al., 2018), and reduce heavy metal contents within the tissues (Xu et al., 2018; Ravanbakhsh et al., 2019; Tanwir et al., 2021), leading to more sustainable agricultural practices. A plant host harbors a mass of bacterial species inside its tissues that impacts plant growth and health (Hardoim et al., 2015). It is well documented that endophytic bacteria can colonize the roots and activate a series of sophisticated mechanisms for aiding plant adaptation to harmful conditions (Lugtenberg and Kamilova, 2009). Several endophytic bacteria can improve plant growth and survival under adverse stresses via the modulation of hormone-related signaling pathways (Oleńska et al., 2020). Mounting evidence has indicated that microbe-mediated changes in hormonal status in plants are primarily attributable to microbial synthesis (e.g., abscisic acid and auxin), degradation (1-aminocyclopropane-1-carboxylate deaminase-mediated ethylene metabolism) of hormones, and alterations in hormone metabolism by bacterial volatile compounds (Bal et al., 2013; Sharifi and Ryu, 2018; Xu et al., 2018). However, it thus far remains unclear how the endophyte-derived signals control the host hormone metabolic pathways for alleviating Cd toxicity.

In this study, we explored the impacts of root endophytic bacteria on tomato adaptation to Cd stress. Among these bacterial isolates, the acetic acid-producing bacteria *Lysinibacillus fusiformis* greatly elevated the capability of the tomato plants to ameliorate Cd toxicity. Transcriptomic, elemental and pharmacological analyses were further combined to elucidate the mechanisms of the endophyte-mediated detoxification of Cd in plants. We found that the interactions between *L. fusiformis* and the tomato roots activated JA signaling pathways to repress the entry of Cd into the roots. Therefore, our study provided a new avenue for exploiting acetic acid-producing endophytes to steer host JA signaling pathways for impeding Cd uptake.

## Materials and Methods

### Isolation of Endophytic Bacteria From Cd-Treated Tomato Roots

Root samples were harvested from 2 months-old tomato plants cultivated in Cd-polluted soils (100 mg Cd kg^–1^ soil). Approximately 1.0 g of roots was placed into 50 mL of plastic tube with sterile water for ultrasonic cleaning for 15 min, followed by sterilization with 1% NaClO for 5 min, 75% alcohol for 2 min and rinsing five times with sterile water. The nutrient agar (NA) plates coated by the last rinsed water were used as a control. The sterilized roots were ground with 15 mL of 0.2 M phosphate buffered saline (PBS) solution and allowed to stand for 15 min. After that, 1 mL of the supernatant was serially diluted and spread on the NA agar plates containing 20 mg L^–1^ CdCl_2_ for 72 h. Bacterial colonies were randomly picked and purified, and a total of 36 isolates were obtained. Bacterial genomic DNA was extracted for amplifying and sequencing 16S rRNA genes.

To evaluate the effects of bacterial isolates on the Cd-stressed tomato plants, a high-throughput screening test was designed. Briefly, tomato seeds were sterilized with 1% NaClO for 10 min and then washed with sterile water. Subsequently, these seeds were cultured on 1/2 Hoagland’s medium for 10 days (d) and then placed on 0.6% agar plates. Each root was incubated with 20 μL of bacterial inoculum at 5 × 10^7^ CFU mL^–1^ for 48 h at 25°C in the dark, and then transferred to the soil (pH 6.5, organic matter 16.7 g kg^–1^, total N 1.25 g kg^–1^, available P 8.9 mg kg^–1^; available K 102.2 mg kg^–1^) under a photoperiod of light (16 h)/dark (8 h) at 25°C. Before transplantation, the soil was saturated with the solution of CdCl_2_ to achieve 100 mg Cd kg^–1^ soil for 2 months and was then autoclaved at 120°C for 1 h before use. The values of maximal PSII photochemical efficiency (Fv/Fm), soil plant analysis development (SPAD), and shoot fresh weight (SFW) were measured for assessing the Cd resistance of plants after 2 weeks of exposure to Cd stress.

### Qualitative Analyses of Acetic Acid-Producing Bacteria, Ultraviolet Mutagenesis and Measurement of Root Acetic Acid Levels and Rhizospheric Organic Acids

Bacterial strains were cultured in basic medium (pH 6.8; 1% yeast extract, 1% glucose, and 3% ethanol) at 30°C for 96 h, followed by centrifugation at 8,000 × g for 15 min. Subsequently, 5 mL of the supernatant was neutralized with 0.1 M NaOH, followed by addition of 20 μL of 5% FeCl_3_. The mixture was heated for 10 min at 100°C. The formation of reddish brown precipitates indicated the presence of acetic acid. To generate acetic acid-deficient strains, wild-type (WT) strains were mutated by ultraviolet radiation and then spread on the agar plates (1% yeast extract, 1% glucose, 2% CaCO_3_, and 1.5% agar) at 28°C for 48 h. The mutated strains that did not produce a transparent zone indicated an inability to produce acetic acid for dissolving CaCO_3_. Furthermore, to measure the acetic acid content, 1.0 g of root tissue was homogenized and centrifuged at 12,000 × g for 15 min at 4°C. Then, acetone was used to dilute the supernatant for acetic acid quantification by gas chromatography-mass spectrometry (GC-MS) (Kim et al., 2017). In addition, root-released organic acids were detected using high performance liquid chromatography (HPLC) as reported by Pii et al. (2015).

### Pot and Split-Root Experiments

For the pot experiments, 10-d-old tomato roots were incubated with 20 μL of cell suspension of *L. fusiformis* Cr33 at 5 × 10^7^ CFU mL^–1^ as described above. These plants were then transplanted to plastic pots filled with the Cd-polluted soil (100 mg Cd kg^–1^ soil). To conduct split-root assays, 3-weeks-old tomato roots were placed into split-root boxes containing 1/2 Hoagland’s medium as reported by Zhou et al. (2019a). In split-root systems, left side of each root box was supplied with bacterial suspensions, while the other side was not. After 3 d of culture, these plants were transferred into the split-root system containing 100 μM CdCl_2_. The medium in the root box was replaced every 3 d. In addition, the root colonization of *L. fusiformis* Cr33 was quantified by quantitative real-time PCR (qRT-PCR) with a pair of gene-specific primers for amplifying the 16S rRNA gene fragment of *L. fusiformis* (F: 5′-ACGGTTTCGGCTGTCGCTAT-3′; R: 5′-TTCCCTACTGCTGCCTCCC-3′).

### Determination of Metal Content and *in situ* Localization of Cd

To measure the total metal content, plant tissues were washed and dried for 24 h at 80°C, followed by treatment with HNO_3_/HClO_4_ (4:1, v/v) and dilution with deionized water as described previously by Lei et al. (2014). The soluble Fe content was detected according to Cassin et al. (2009). Briefly, plant tissues were ground with deionized water and then centrifuged at 12,000 × g for 15 min. The supernatant was used to measure soluble Fe content. The contents of total Fe and Cd, and soluble Fe were quantified via inductively coupled plasma-atomic emission spectroscopy (ICP-AES). For measuring apoplastic Fe content, the roots were serially treated with CaSO_4_, 2.2-bipyridyl, N_2_ and Na_2_S_2_O_4_ according to Jin et al. (2007). The collected solutions were used to determine root apoplastic Fe via detection of the Fe^2+^-bipyridyl complex at 520 nm.

*In situ* localization of Cd was detected in the roots as described by Balestri et al. (2014). Briefly, excised roots were rinsed with deionized water and then immediately immersed in dithizone working solution containing 30 mg dithizone, 20 mL deionized water and 60 mL acetone for 2 h. The roots with reddish precipitates were washed with deionized water and then photographed. Additionally, the availability of Fe and Cd in the rhizospheric soil was extracted and analyzed by ICP-AES according to Chen et al. (2015).

### RNA Sequencing (RNA-Seq) and qRT-PCR Analyses

Three-week-old tomato plants grown in hydroponic systems were subjected to treatment with or without bacterial suspension for 48 h. These plants were then transferred into the split-root systems containing 100 μM CdCl_2_. After that, the root tissues were harvested and immediately ground in liquid nitrogen. Then, total RNA from the root samples was extracted using TRIzol reagent (Invitrogen, United States) according to the manufacturer’s instructions. The RNA samples from three biological repeats were analyzed using an Agilent 2100 Bioanalyzer and then used for RNA sequencing through the Illumina HiSeq 4000 platform (Illumina, United States). Clean reads were mapped to the reference genome sequence of *Solanum lycopersicum* Heinz 1706, and then submitted to the NCBI SRA database (No. PRJNA695320). The R package DEGseq was used to screen differentially expressed genes (DEGs) at *P* ≤ 0.05 and a value of log_2_ fold change > 1.0 or < −1.0. Gene Ontology (GO) enrichment analyses of the DEGs were screened at a cutoff of FDR ≤ 0.05 using the GOseq R package (Chen et al., 2005). Kyoto Encyclopedia of Genes and Genomes (KEGG) pathway analyses were conducted using the KOBAS software (Mao et al., 2005).

For the qRT-PCR analyses, total RNA samples were extracted and used as qPCR templates. The qRT-PCR reactions were performed using the SYBR Green qPCR Master Mix (Takara, Japan) in an Applied Biosystems^TM^ 7500 Real-Time PCR system. The tomato *Actin* gene was used as an internal control. The primers used for qRT-PCR analyses were reported recently by Zhou et al. (2019b).

### Detection of Root Ferric Chelate Reductase (FCR) Activity and 2,3,5-Triphenyltetrazolium Chloride (TTC) Assays

Root FCR activities were measured as described by Grusak (1995). Briefly, whole roots were incubated in the assay solution (0.1 mM Fe-EDTA, 0.1 mM FerroZine, 0.5 mM CaSO_4_, 0.1 mM bathophenanthroline-disulfonic; pH 5.5) for 2 h at 28°C. Absorbance of the solutions was determined at 562 nm and the formation of Fe^2+^-FerroZine was detected using an extinction coefficient of 27.9 mm^–1^ cm^–1^. To measure cell viability, tomato roots were incubated in 15 mL of assay solution (0.4% TTC in 60 mM PBS, pH 7.0) for 6 h at 37°C. Reduced TTC was extracted with ethanol and the absorbance was recorded at 485 nm (Hawrylak-Nowak et al., 2015).

### Analyses of Physiological Indexes, Root JA and NO Content and Ultrastructural Observation

Chlorophyll content was assessed on the fully expanded leaves based on the analysis of the SPAD values using a portable chlorophyll meter as previously reported by Lei et al. (2014). The values of Fv/Fm and actual PSII photochemical efficiency (ΦPSII) were determined using a FluorCam 7 system (Zhou et al., 2019a). The chlorophyll levels were analyzed based on the method described by Porra (2002). Briefly, leaf tissues were immersed in 80% acetone and incubated for 2 d in the dark. Absorbance of the extracted solutions was measured at 645 and 663 nm. The levels of chlorophyll were assessed using the formula: 20.21 × A645 + 8.02 × A663. In addition, H_2_O_2_ content, electrolyte leakage (EL), and malondialdehyde (MDA) values were measured as described by Wang et al. (2010). Root JA content was measured as reported by Kim et al. (2017). Briefly, about 200 mg of fresh tomato roots were ground into the powder using liquid nitrogen and then combined with 2mL of extraction solution (methanol: formic acid:water = 15:1: 4), followed by centrifugation at 12,000 × g for 30 min. The supernatant was loading into a Sep-Pak C18 column and then eluted with the extracted solution. The collected solution was evaporated and then dissolved in 200 μL of 80% methanol for analyzing JA content using HPLC. In addition, root NO content was quantified by the oxyhemoglobin-based spectrophotometric assay as described recently by Zhou et al. (2019b). To observe chloroplast ultrastructure, leaf tissues were cut into small pieces and immediately fixed with 1% glutaraldehyde, and then subjected to gradient dehydration with acetone. Finally, the leaf samples were embedded in EPON 812 resin and cut into sections (70–100 nm) for ultrastructural observations.

### Statistical Analysis

The experimental data were analyzed by SPSS 10.0 software (SPSS Inc., Chicago, United States) with using Student’s *t*-test or Duncan’s multiple range tests with one-way analysis of variance at *P* < 0.05.

## Results

### Screening of Root Endophytic Bacteria With Cd-Detoxifying Properties in Plants

**FIGURE 1:**
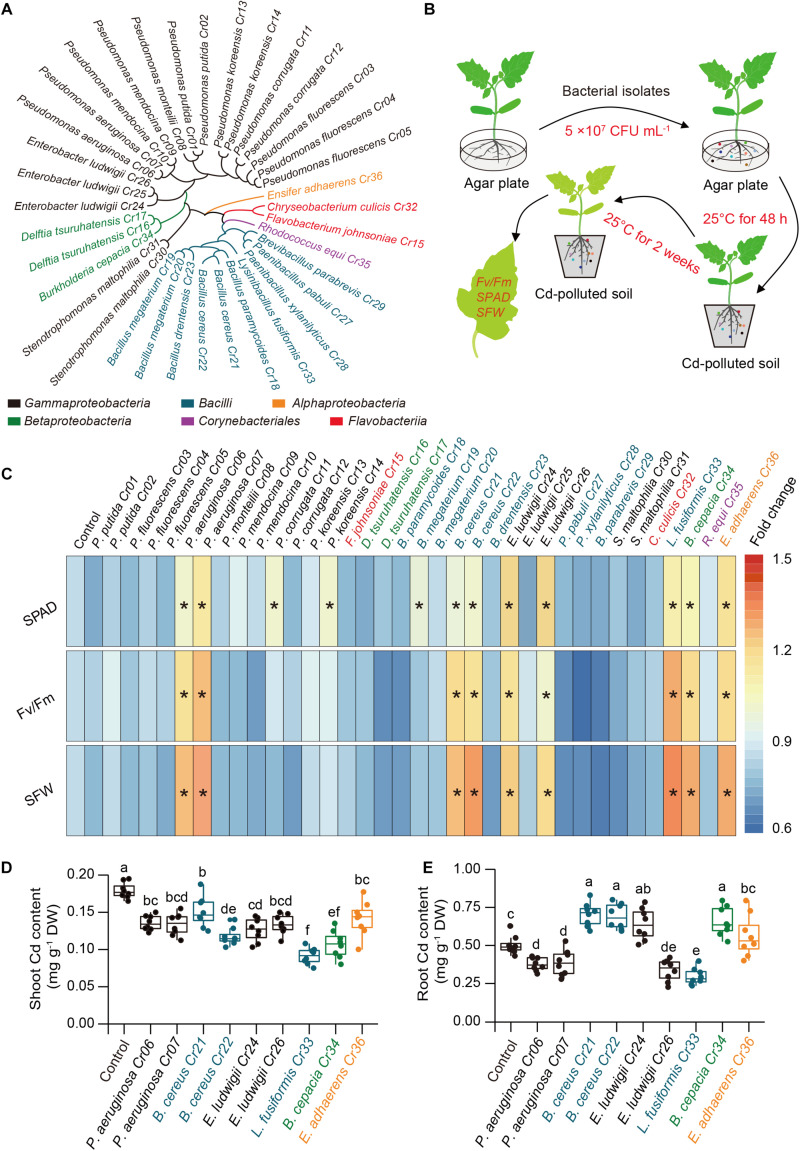
Effects of soil drench with endophytic bacteria on Cd resistance in tomato plants. **(A)** Taxonomic cladogram of bacterial isolates. **(B)** High-throughput assays of Cd-detoxifying bacterial isolates. Ten-day-old tomato roots were co-cultured with or without bacterial suspension on agar plate at 25°C in the dark for 48 h. the non-inoculated (control) and inoculated plants were then transplanted into Cd-contaminated soils (100 mg Cd kg^–1^ soil) at 25°C. After 2 weeks of culture, the values of Fv/Fm, SPAD and SFW were determined for assessing the ability of plants to tolerate Cd stress. **(C)** Heatmap analysis for Fv/Fm, SPAD and SFW. Asterisks indicated significant differences between the control and inoculated plants (*n* = 8 biological replicates) using Student’s test at *p* < 0.05. **(D)** Shoot and **(E)** root Cd content. Different letters indicated significant differences among different bacterial strain-inoculated plants (*n* = 8 biological replicates) using Duncan’s multiple range test at *p* < 0.05.

A total of 36 endophytic bacteria strains were isolated from the roots of 2-month-old tomato plants cultivated in soil polluted with about 100 mg Cd kg^–1^ soil. Bacterial isolates were identified by PCR amplification of 16S rRNA genes, and the sequences were assigned to species using the NCBI blast tool (Supplementary Table 1). Based on 97% sequence similarity for the 16S rRNA genes, these bacterial strains were assigned to six phylogenetic taxa at the class level, including *Alpha*-, *Beta- and Gamma-proteobacteria, Bacilli, Corynebacteriales*, and *Flavobacteria* (Figure 1A). Furthermore, these isolates were selected to assess their potential for detoxifying Cd in the tomato plants. Using high-throughput screening assays (Figure 1B), nine bacterial strains were found to distinctly mitigate Cd stress in the tomato plants, as reflected by the higher values of Fv/Fm, SPAD and SFW, which were used as a proxy assessment for Cd toxicity in plants (Figure 1C). Compared with the non-inoculated (control) plants, soil drenched with the nine isolates significantly reduced shoot Cd content, whereas some of the isolates increased the root Cd content (Figures 1D,E). Among these isolates, *L. fusiformis* Cr33 exhibited the greatest ability to relieve Cd stress and inhibit plant uptake of Cd, and was thus selected for the subsequent experiments.

### Inoculation With *L. fusiformis* Alleviated Cd Stress and Reduced Cd Uptake in Tomato

After 4 weeks of Cd treatment, serious chlorosis was observed in new leaves with a marked reduction in chloropyll content and Fv/Fm values, but this was clearly relieved by supplying the soil with *L. fusiformis* Cr33 (Supplementary Figures 1A–C). The Cd content was notably lower in the shoots and roots of the inoculated plants than the non-inoculated (control) plants (Supplementary Figure 1D). Within 4 weeks of inoculation, the population of *L. fusiformis* Cr33 had abundantly colonized the roots within 7 d (0.3–1.2 × 10^7^ CFU g^–1^), following which it decreased (0.4–3.5 × 10^6^ CFU g^–1^) (Supplementary Figure 1E).

**FIGURE 2:**
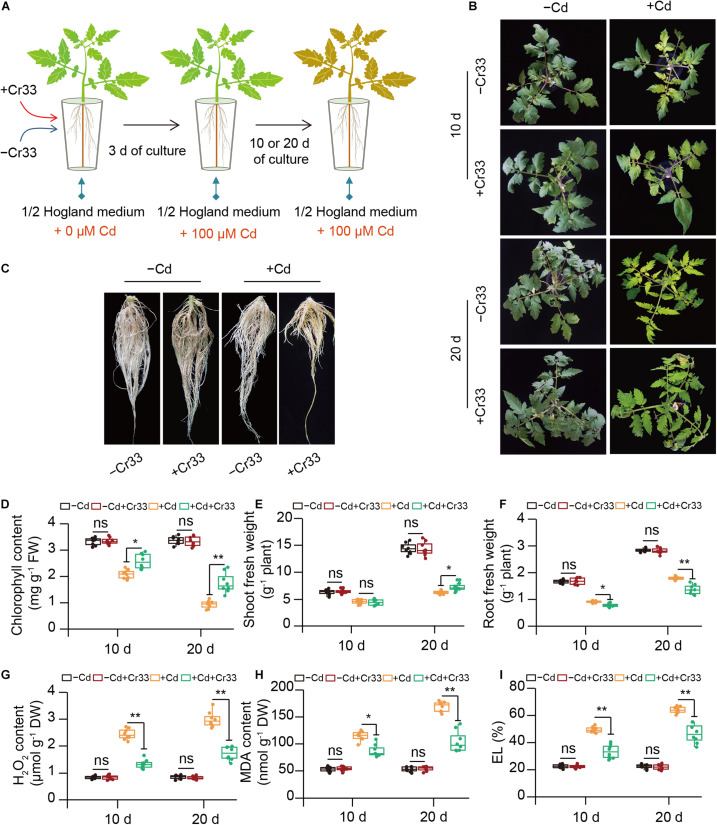
Root inoculation with *L. fusiformis* Cr33 ameliorated Cd stress in tomato plants. **(A)** Three-weeks-old tomato plants cultured in the split-root systems were inoculated with or without Cr33 for 3 d. Then, these plants were exposed to 0 or 100 μM Cd with or without Cr33 for 10 and 20 d, respectively. These plants were used to analyze shoot phenotypes **(B)**, root growth after 20 d of culture **(C)**, chlorophyll content **(D)**, shoot **(E)** and root **(F)** fresh weight, H_2_O_2_ content **(G)**, and MDA **(H)** and EL **(I)**. Asterisks indicated significant differences between the control and inoculated plants (*n* = 8 biological replicates) using Student’s test (ns, not significant; *p < 0.05; **p < 0.01).

**FIGURE 3:**
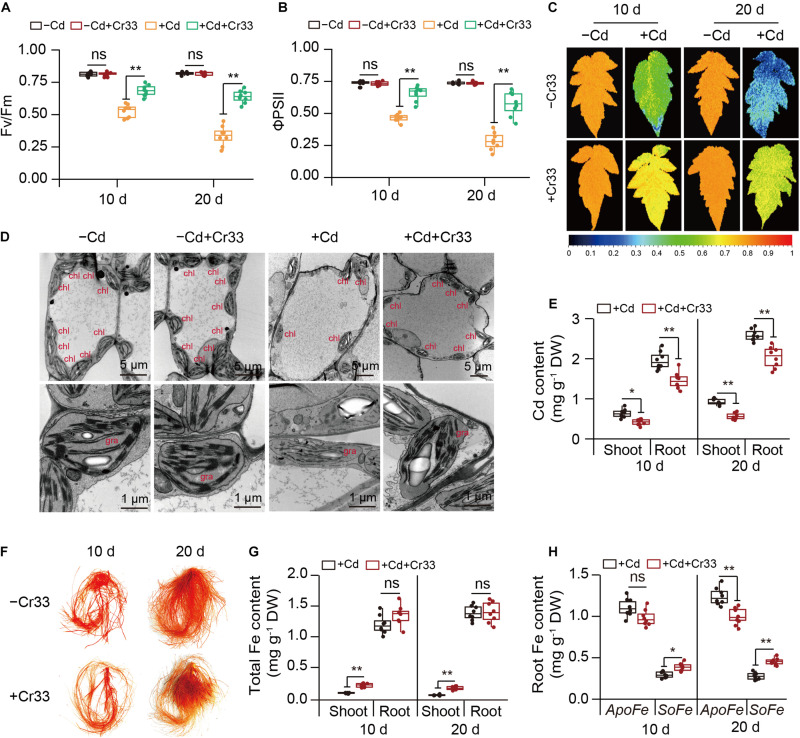
Root inoculation with *L. fusiformis* Cr33 reduced Cd toxicity in tomato plants. Three-weeks-old tomato plants cultured in the split-root systems were inoculated with or without Cr33 for 3 d. Then, these plants were exposed to 0 or 100 μM Cd with or without Cr33 for 10 and 20 d, respectively. These plants were used to analyze Fv/Fm **(A)**, ΦPSII **(B)**, Fv/Fm images **(C)**, chloroplast ultrastructure (chl, chloroplast; gra, grana) **(D)**, shoot and root Cd content **(E)**, root Cd localization **(F)**, total Fe content **(G)**, root apoplastic (*ApoFe*) and soluble Fe (*SoFe*) **(H)**. Asterisks indicated significant differences between the control and inoculated plants (*n* = 8 biological replicates) using Student’s test (ns, not significant; *p < 0.05; **p < 0.01).

In the split-root systems, 3-weeks-old tomato roots were treated with or without cell suspension of Cr33 (Figure 2A). Cd stress resulted in chlorotic symptoms with a reduction in chlorophyll levels and biomass (Figures 2B–F). However, root inoculation with Cr33 inhibited root growth compared with the non-inoculated (control) plants under Cd stress conditions (Figures 2C,F). The levels of H_2_O_2_ were distinctly increased in the leaves of the Cd-treated plants, whereas the Cr33-inoculated leaves accumulated less H_2_O_2_ levels than the control plants (Figure 2G). Accordingly, the inoculated plants displayed lower the values of MDA and EL in the leaves than the control plants (Figures 2H,I). Additionally, Cd exposure led to marked decreases in the values of Fv/Fm and ΦPSII, whereas their values were remarkably lower in the control plants than the inoculated plants under Cd stress conditions (Figures 3A–C). However, there was no obvious difference in photosynthesis between the control and inoculated plants under non-Cd condition (Figures 3A–C). Cd stress also led to a marked reduction in chloroplast number, swollen chloroplasts and fewer granum lamellae, but this was largely alleviated by root inoculation with Cr33 (Figure 3D). The TTC cell viability assays also showed that the inoculated plants maintained higher root viability than the control plants under Cd stress conditions (Supplementary Figure 2). Moreover, the inoculated plants exhibited less Cd content than the control plants (Figure 3E). Accordingly, *in situ* localization of Cd showed reddish precipitates in the Cd-treated roots, whereas the inoculated roots exhibited less reddish precipitates than the control roots (Figure 3F). The inoculated plants exhibited a reduction of about 50% shoot Fe content under Cd stress compared with the control plants, but no striking difference was observed for root Fe content (Figure 3G). However, the inoculated roots exhibited less apoplastic Fe (*ApoFe*) and higher soluble Fe (*SoFe*) levels than the control plants (Figure 3H).

### *L. fusiformis*-Derived Acetic Acid Increased Cd Resistance in Tomato

**FIGURE 4:**
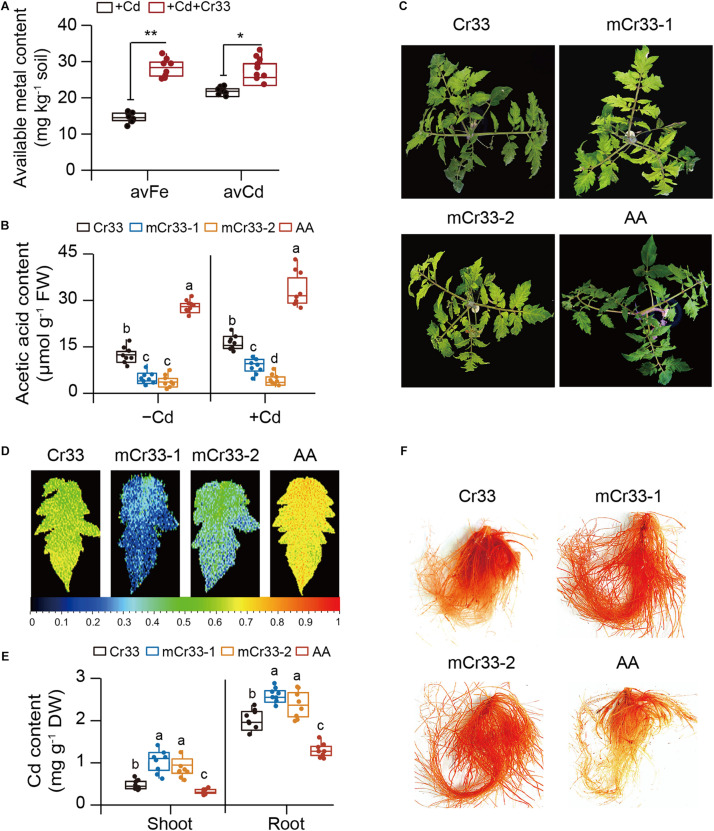
*L. fusiformis* Cr33-released acetic acid conferred the increased availability of rhizospheric Fe and Cd, and Cd resistance in tomato plants. **(A)** The bioavailability of rhizospheric Fe and Cd was quantified after 2 weeks of soil drench with Cr33. Asterisks indicated significant differences between the non-inoculated (control) and inoculated plants (*n* = 8 biological replicates) using Student’s *t*-test (*p < 0.05; **p < 0.01). **(B–F)** Three-weeks-old tomato plants were cultured in the split-root systems containing 100 μM Cd with or without Cr33 or its mutant strains (mCr33-1 and -2) for 20 d. These plants were used to analyze root acetic acid content **(B)**, shoot phenotypes **(C)**, Fv/Fm images **(D)**, shoot and root Cd content **(E)**, and root Cd localization **(F)**. Different letters indicated significant differences among different experimental groups (*n* = 8 biological replicates) using Duncan’s multiple range test at *p* < 0.05.

The bioavailability of Cd and Fe in the rhizosphere is positively related to Cd toxicity in plants (Xu et al., 2018; Wang et al., 2020). We therefore determined their content in rhizospheric soils from both the control and Cr33-inoculated plants. The content of rhizospheric Cd was slightly higher in the bacteria-treated soils than in the untreated soils (Figure 4A). By contrast, the levels of rhizospheric Fe were about twofold higher in the bacteria-treated soils than in the untreated soils. Root-secreted organic acids can influence the availability of nutrient elements and heavy metals in the rhizosphere (Rajkumar et al., 2012; Montiel-Rozas et al., 2016). Thus, the levels of organic acids in the rhizospheric soils were determined. Compared with the control plants, the rhizospheric soils from the inoculated plants had greater total organic acid content. Among these organic acids, the rhizospheric soils from the inoculated plants displayed significantly higher acetic acid levels than the control plants (Supplementary Figure 3). Moreover, qualitative assays revealed that the reaction of Cr33 culture with FeCl_3_ produced reddish brown substances (Supplementary Figure 4), indicating that *L. fusiformis* was an acetic acid-producing bacterium.

As shown in Figure 4B, two acetic acid-deficient mutants (mCr33-1 and -2) did not considerably increase the root acetic acid levels compared with Cr33. Moreover, the Cd-induced chlorotic symptoms and reduction of Fv/Fm values were not largely ameliorated by the mutant strains (Figures 4C,D). The levels of Cd were distinctly higher in the shoots and roots of the mCr33-exposed plants than those of the Cr33-exposed plants (Figure 4E). In line with this, *in situ* localization of Cd showed a notable reduction in reddish precipitates in the roots colonized by Cr33, but not in the mCr33-inoculated roots (Figure 4F). To further confirm whether high-level acetic acid conferred increased Cd resistance in plants, split-root assays were conducted as described above. Acetic acid (AA) treatment markedly alleviated the Cd-induced leaf chlorosis with higher Fv/Fm values and less Cd content (Figures 4C–E). Consistent with this, the reddish precipitates were substantially reduced in the AA-treated roots (Figure 4F).

### Transcriptome Analyses of *L. fusiformis*-Treated Tomato Roots

**FIGURE 5:**
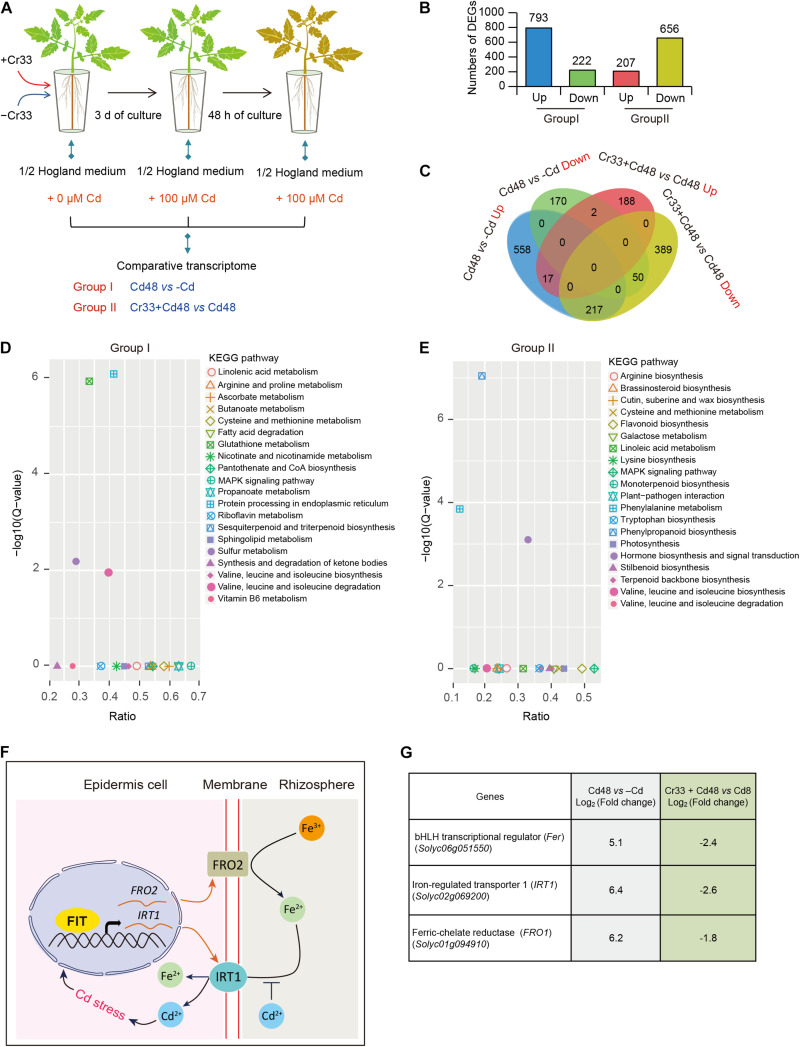
Transcriptome analyses of *L. fusiformis* Cr33-inoculated roots in response to Cd stress. **(A)** Three-week-old tomato plants cultured in the split-root systems were inoculated with or without Cr33 for 3 d. Then, these plants were exposed to 100 μM Cd with or without Cr33 for 48 h. Total RNA samples extracted from the treated roots were used for RNA-Seq analyses, including Group I (Cd48 vs. –Cd) and II (Cr33 + Cd48 vs. Cd48). **(B)** Numbers of DEGs in both Group I and II. **(C)** Venna diagram of shared and specific DEGS between the Group I and II. **(D,E)** KEGG analyses for up-regulated DEGs from the Group I and II. **(F)** A model for the Cd-activated Fe uptake leading to aggravate Cd toxicity in plants. Cd-induced expression of Fe uptake-related genes including *FIT* (a functional homolog of *Fer*), *IRT1*, and *FRO2* resulted in the increased Cd uptake in plants. **(G)** Expression profiles of *Fer*, *IRT1*, and *FRO1* in both the Group I and II.

To probe the molecular mechanisms of *L. fusiformis*-induced Cd resistance of tomato plants, RNA-Seq was performed to examine gene expression profiles of the Cd-treated roots colonized by Cr33. For this, tomato plants cultured in hydroponic systems with or without Cr33 were treated with 100 μM CdCl_2_ for 0 and 48 h (Figure 5A). Gene expression changes in the roots were examined by comparing the control plants (Cd48) with the Cr33-inoculated plants (Cr33 + Cd48). An additional condition evaluation of the impacts of 100 μM Cd^2+^ on the gene expression profiles in the roots was also conducted. A total of 1,015 (Supplementary Table 2) and 863 (Supplementary Table 3). DEGs exhibited significant differential expression in both Group I (Cd48 vs. -Cd) and II (Cr33 + Cd48 vs. Cd48), respectively (Figures 5B,C). The GO enrichment analyses of the DEGs showed that several genes involved in diverse processes such as detoxification and response to stimulus were remarkably induced by Cd stress and Cr33 (Supplementary Figure 5). As shown in Figure 5D, KEGG enrichment pathway analyses for the Group I revealed that Cd stress strikingly impacted several metabolic pathways such as sulfur and glutathione, which are responsible for the detoxification of Cd in plants (Gill and Tuteja, 2011; Han et al., 2020; Xu et al., 2020). Conversely, root inoculation with Cr33 markedly affected several pathways such as phenylalanine metabolism, phenylpropanoid biosynthesis, hormone biosynthesis and signal transduction (Figure 5E).

As shown in Figure 5C, 217 of the 518 DEGs that were greatly induced by Cd stress were down-regulated in the Cr33-inoculated roots, indicating that a mass of Cd-responsive genes in the roots was significantly repressed by Cr33 (Supplementary Table 4). Previously, the Cd-induced expression of Fe uptake-associated genes was shown to promote Cd uptake and enhance Cd toxicity in *Arabidopsis* plants (Figure 5F). Among the shared DEGs, the transcription levels of Fe uptake-associated genes including *Fer*, *FRO1*, and *IRT1* were considerably increased in the Cd-exposed roots, whereas root inoculation with Cr33 significantly down-regulated their expression (Figure 5G). Additionally, several genes associated with the biosynthesis of JA and signal transduction were observably activated in the Cd-treated roots colonized by Cr33 (Supplementary Table 5).

### *L. fusiformis* Inhibited Root NO Burst and Fe Deficiency Response Under Cd Stress

**FIGURE 6:**
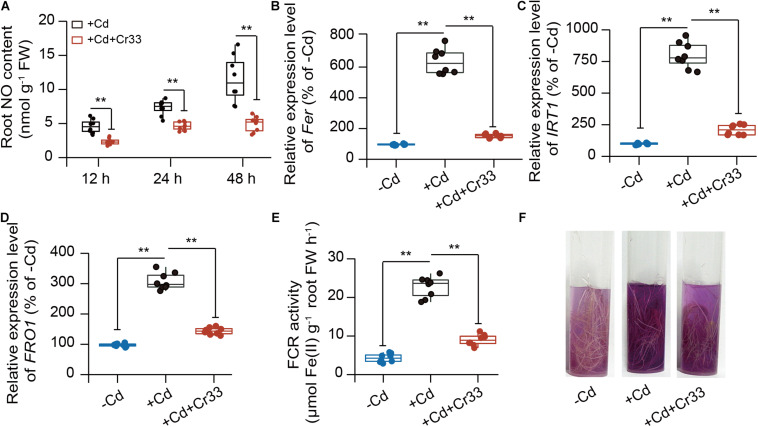
Root inoculation with *L. fusiformis* Cr33 inhibited root Fe deficiency responses under Cd stress condition. Three-weeks-old tomato plants cultured in the split-root systems were inoculated with or without Cr33 for 3 d. Then, these plants were exposed to 100 μM Cd with or without Cr33 for 48 h. These plants were used to analyze root No content **(A)**, the expression of *Fer* **(B)**, *IRT1* **(C)**, *FRO1* **(D)**. In addition, root FCR activities were quantified **(E)** and assessed by observing purple color **(F)**. Asterisks indicated significant differences between different experimental groups (*n* = 8 biological replicates) using Student’s *t*-test (**p < 0.01).

As shown in Figure 6A, Cd stress triggered the production of NO in the roots, whereas root NO burst was dramatically restrained in the Cr33-inoculated roots. Since NO is essential for activating the expression of the *FER*, *IRT1*, and *FRO1* genes in tomato roots under Fe deficiency (Graziano and Lamattina, 2007; Chen et al., 2015), we evaluated the effects of Cr33 on root Fe deficiency responses in the Cd-exposed plants. Their transcripts were quantified by qRT-PCR in the roots after 48 h of exposure to Cd stress. The expression of the *FER*, *IRT1*, and *FRO1* genes was relatively higher in the Cd-treated roots than the untreated roots, but root inoculation with Cr33 reduced their transcripts (Figures 6B–D). The FCR activities in the Cd-treated roots were remarkably induced by Cd stress, whereas inoculation with Cr33 strikingly repressed root FCR activities (Figures 6E,F). This decline of FCR activities was in concert with the reduced expression of *FRO1* (Figure 6D).

### JA Signals Were Required for the *L. fusiformis*-Alleviated Cd Toxicity of Tomato

**FIGURE 7:**
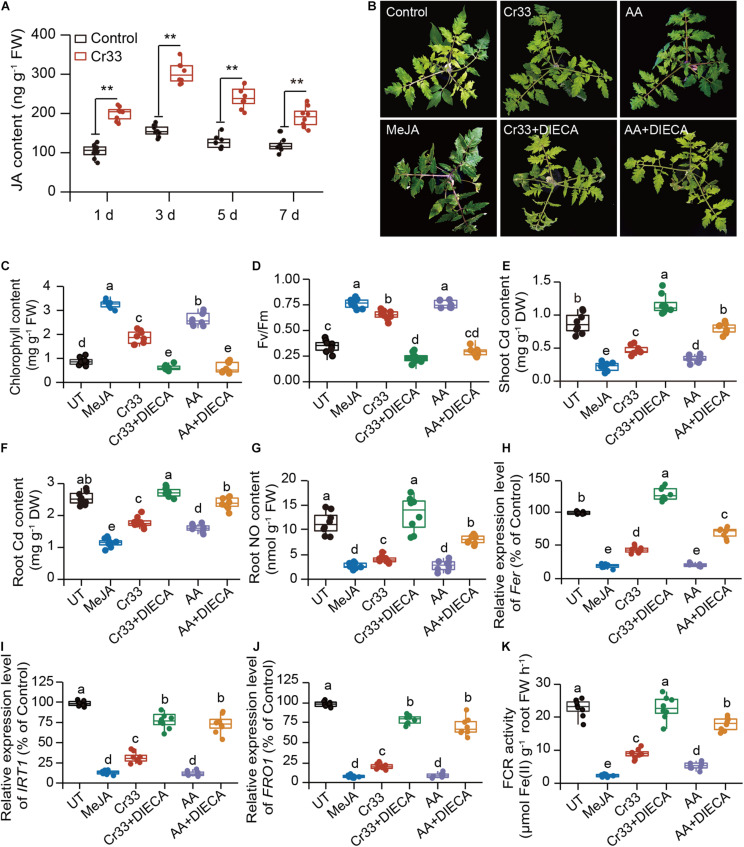
Involvement of JA signals in the *L. fusiformis* Cr33-induced Cd resistance in tomato plants. **(A)** Root JA content within 7 d of Cd treatment. Asterisks indicated significant differences between the non-inoculated (control) and inoculated plants (*n* = 8 biological replicates) using Student’s *t*-test (**p < 0.01). Three-weeks-old tomato plants were exposed to 100 μM Cd with or without cell suspensions of Cr33 at the final density of 5 × 10^7^ CFU mL^–1^, 0.15 mM acetic acid (AA), 0.05 mM MeJA, Cr33 plus 0.2 mM DIECA (Cr33 + DIECA), and 0.15 mM AA plus 0.2 mM DIECA (AA + DIECA) for 20 d. The untreated (UT) and treated plants were used to analyze shoot phenotypes **(B)**, chlorophyll content **(C)**, Fv/Fm values **(D)**, shoot **(E)** and root **(F)** Cd content, root NO content **(G)**. qPCR analyses of the expression of *Fer* **(H)**, *IRT1* **(I)** and *FRO1* **(J)**, and root FCR activity **(K)** after 48 h of treatments. Different letters indicated significant differences among different experimental groups (*n* = 8 biological replicates) using Duncan’s multiple range test at *p* < 0.05.

High-level acetic acid promotes *de novo* JA synthesis and further activates the JA signaling pathway for improving drought tolerance in plants (Kim et al., 2017). For this reason, we assessed the effects of *L. fusiformis* Cr33 on the JA content in the Cd-exposed tomato roots. Compared with the non-inoculated (control) plants, the JA levels were remarkably increased in the Cr33-inoculated roots after 1 d of exposure to Cd stress and thereafter stabilized at a higher level over 7 d (Figure 7A). It was thus possible that the Cr33-induced increases in JA levels contributed to reducing the Cd toxicity in the plants. To validate this hypothesis, the Cd resistance of plants treated with methyl jasmonate (MeJA) was also evaluated. MeJA treatment strikingly relieved Cd stress in plants, as reflected by the higher chlorophyll content, Fv/Fm values, and lower Cd levels (Figures 7B–F).

After 48 h of Cd treatment, root NO content was relatively lower in the MeJA-treated plants than the untreated (UT) plants, which was similar to the observation for the Cr33-inoculated roots (Figure 7G). Diethyldithiocarbamic acid (DIECA), a JA biosynthetic inhibitor, was applied to the Cd-treated plants colonized by Cr33. Root inoculation with Cr33 did not relieve Cd toxicity in the DIECA-treated plants, which displayed serious leaf chlorosis, lower chlorophyll levels and Fv/Fm values, and greater Cd content (Figures 7B–F). Similarly, DIECA treatment largely weakened the AA-alleviated Cd toxicity in the plants. Treatment with Cr33 or MeJA inhibited the transcription of *Fer*, *FRO1* and *IRT1*, and the activities of FCR. Conversely, DIECA treatment enhanced root Fe deficiency responses in both the Cr33- and AA-treated plants (Figures 7H–K). In addition, the root NO content was remarkably increased in both the Cr33- and AA-treated plants when treated with DIECA (Figure 7G).

## Discussion

Remediating soil Cd contamination is costly and challenging, and thus there is an increasing demand for alternative strategies to address Cd pollution in agricultural soils (Pulford and Watson, 2003). Steering plant hormone metabolism toward the repression of Cd uptake may preserve food safety and improve plant performance under Cd-polluted conditions (Lei et al., 2017). Herein, we explored the impacts of the acetic acid-producing endophytic bacteria *L. fusiformis* on the plant hormone JA, which negatively regulates Fe uptake and translocation (Lei et al., 2020). The endophyte-derived acetic acid stimulated the JA biosynthesis and further suppressed the root Fe deficiency responses imposed by Cd stress, which contributed to reduction of Cd accumulation. Hence, our findings suggested that manipulation of host JA signals by root endophytic bacteria effectively prevented Cd absorption in the plants.

Cd stress often results in the reduction of plant photosynthesis, biomass and root viability (Hawrylak-Nowak et al., 2015; Xu et al., 2018; Pan et al., 2019). In accordance with previous reports on the harmful impacts of Cd stress, the values of photosynthetic parameters were largely decreased in the Cd-exposed leaves. Compared with the control plants, these photosynthetic indexes were observably higher in the *L. fusiformis*-inoculated plants under Cd stress. Observations of the photosynthetic apparatus further showed that the chloroplast structures were abnormal and that there were fewer chloroplasts and stacked grana in the Cd-exposed leaves. However, root inoculation with *L. fusiformis* mitigated the toxicity of Cd to the photosynthetic system. Cd stress also caused a considerable reduction in root viability, while the inoculated roots displayed higher viability than the control plants. Therefore, these findings indicated that the inoculated plants experienced fewer of the toxic effects imposed by Cd stress.

The bioavailability of Cd in the soil is an important factor that affects Cd uptake in plants (Rajkumar et al., 2012; Xu et al., 2018). We found that inoculation with *L. fusiformis* increased the bioavailability of Cd in the rhizospheric soils. This raised a question why higher Cd availability did not aggravate the Cd toxicity in the tomato plants. Herein, we also found that the bioavailability of Fe in the rhizospheric soils was remarkably increased in the *L. fusiformis*-treated soils compared with the untreated soils. In the hydroponic experiments, root inoculation with *L. fusiformis* also promoted root cell wall Fe remobilization, thereby increasing its availability. Wang et al. (2020) reported that the Cd-tolerant bacteria *Burkholderia* sp. Y4 prevents Cd uptake in rice by increasing the bioavailability of micronutrients such as Fe and Mn in the rhizosphere soils. Cd frequently competes with Fe for the absorption sites and inhibits Fe uptake, thereby provoking Fe deficiency responses (Wu et al., 2012). The increased Fe source can effectively alleviate the Cd toxicity in plants by repressing the root uptake of Cd (Wu et al., 2012; Sebastian and Prasad, 2016; Chen et al., 2017). Hence, the increased bioavailability of Fe by *L. fusiformis* reinforced the competitiveness of Fe with Cd for the absorption sites and further inhibited the entry of Cd into the root cells, which at least partially contributed to less Cd accumulation in the plants.

In this study, soil drenched with *L. fusiformis* led to higher acetic acid levels in the rhizospheric soil compared with the control plants. Qualitative assays of acetic acid further indicated that *L. fusiformis* was an acetic acid-producing endophytic bacterium. It is increasingly evidenced that high-level organic acid such as acetic and malic acid can facilitate plant tolerance to adverse conditions such as drought, aluminum (Al) and Cd stress (Hawrylak-Nowak et al., 2015; Pii et al., 2015; Kim et al., 2017). Hence, higher acetic acid levels in the *L. fusiformis*-inoculated roots may be conducive to alleviating Cd toxicity in tomato plants. To verify this hypothesis, we investigated the impacts of acetic acid-deficient strains on the Cd-tolerating capacity of the tomato plants. We found that inoculation with acetic acid-deficient strains failed to mitigate Cd stress in the tomato plants. Furthermore, exogenous acetic acid distinctly elevated the capability of plants to tolerate Cd stress. These results strongly supported a pivotal role of acetic acid in detoxifying Cd in plants. However, the molecular mechanisms of the acetic acid-mediated alleviation of Cd toxicity in plants have remained elusive thus far.

Besides the increased Fe availability, *L. fusiformis* may initiate alternative complementary pathways for relieving Cd toxicity in plants. More recently, increased acetic acid level can induce the biosynthesis of JA and thus improve plant drought resistance (Kim et al., 2017). Herein, the biosynthesis of JA in plants was substantially induced by *L. fusiformis*. Similar results were also observed for the acetic acid-treated tomato plants. We further monitored the responses of *L. fusiformis*-inoculated plants to Cd stress in the presence of the JA biosynthetic inhibitor DIECA. It was clearly observed that *L. fusiformis* failed to relieve Cd toxicity in the DIECA-treated plants, indicating that the enhanced JA synthesis was responsible for the *L. fusiformis*-induced Cd resistance in the plants. In fact, Cd toxicity is mainly as a consequence of the dysfunction of essential element absorption, especially Fe, since Cd stress induces leaf chlorosis and the molecular responses resembling those triggered by Fe deficiency (Clemens, 2006; Wu et al., 2012; Sebastian and Prasad, 2016; Chen et al., 2017). Fe deficiency often stimulates the production of NO, which is essential for enhancing the transcription of Fe uptake-related genes in plants (Graziano and Lamattina, 2007; Zhou et al., 2019b). Cd stress can induce root NO burst in many plant species such as *Arabidopsis* and wheat (Bartha et al., 2005; Valentovicova et al., 2010; Arasimowicz-Jelonek et al., 2011; Lei et al., 2020). Nevertheless, overproduction of NO in the roots intensifies the Cd toxicity in *Arabidopsis* plants, which is positively related to the enhancement of Cd uptake (Besson-Bard et al., 2009). Herein, Cd stress considerably triggered NO burst in the tomato roots within several hours, whereas the *L. fusiformis*-inoculated plants exhibited less root NO accumulation. In accordance with this, the expression of Fe uptake-related genes was greatly weakened in the *L. fusiformis*-inoculated roots under Cd stress compared with the control plants. Reductions in *IRT1* mRNA transcripts have been reported to interdict root uptake of Cd (Zhu et al., 2012). Thus, the suppression of root NO burst by *L. fusiformis* effectively minimized the activation of *IRT1* expression in plants, thereby reducing Cd accumulation. However, treatment with DIECA almost abolished the *L. fusiformis*-mediated inhibition of root NO burst in the Cd-treated plants. It has recently been indicated that exogenous JA reduces root NO levels in the Cd-stressed plants, thereby down-regulating the expression of *AtIRT1* (Lei et al., 2020). Consistently, the Cd-stressed tomato plants exhibited lower root NO levels and *IRT1* expression after JA treatment. Hence, our results suggested that the JA-mediated repression of NO signals was responsible for the *L. fusiformis*-inhibited Cd uptake in the plants.

## Conclusion

**FIGURE 8:**
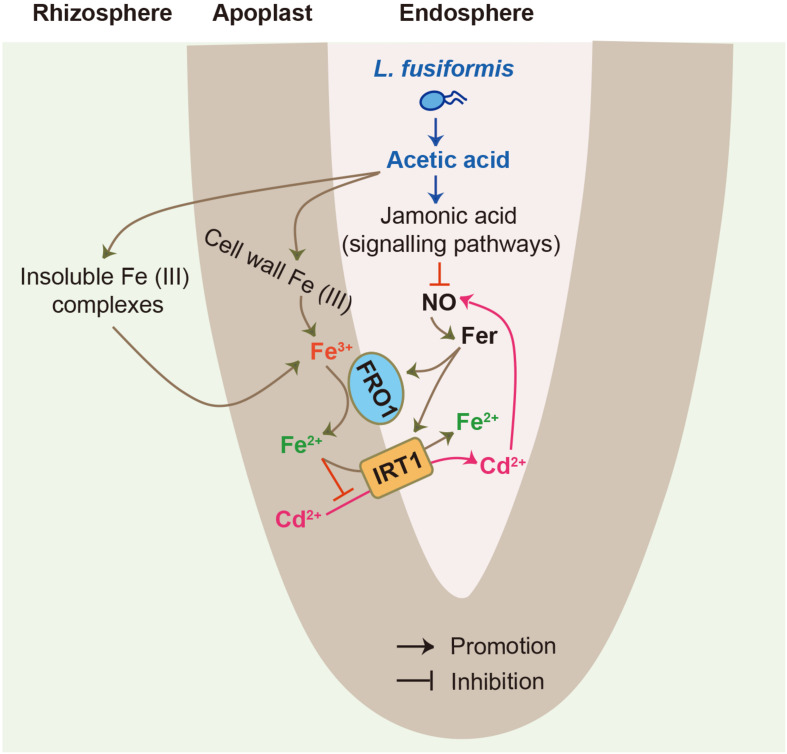
A model for illustrating root endophytic bacteria *L. fusiformis*-mediated Cd detoxification in tomato plants. *L. fusiformis*-released acetic acid led to the increased bioavailability of rhizospheric and cell wall Fe. Moreover, high-level acetic acid also activated the JA signaling pathway to suppress root NO burst, thereby weakening Cd-induced Fe deficiency responses. These synergistic effects contributed to inhibit the IRT1-mediated Cd uptake in plants.

In summary, although endophytic bacteria have previously been reported to improve plant health and survival under adverse conditions, the mechanisms underpinning these beneficial services have been sparsely explored. Herein, an illustrated model was provided for endophyte-mediated Cd detoxification in tomato plants (Figure 8), in which the *L. fusiformis*-derived acetic acid resulted in increases in Fe bioavailability and acetic acid levels in the roots. Moreover, high-level acetic acid provoked the JA signaling pathway to inhibit root NO burst, thus attenuating the root Fe deficiency responses imposed by Cd stress. Consequently, these synergistic effects contributed to hampering the entry of Cd into root cells and thus mitigating Cd toxicity in plants: (1) the increased Fe bioavailability enhanced the competitiveness of Fe with Cd for metal transporters such as IRT1; and (2) the repression of NO signals down-regulated Fe uptake-related genes under Cd stress conditions, thereby inhibiting the IRT1-mediated Cd uptake. Therefore, our findings provide novel insights into the mechanisms of endophyte-mediated Cd detoxification in plants.

## Data Availability Statement

The original contributions presented in the study are publicly available. This data can be found here: NCBI SRA database (No. PRJNA695320).

## Author Contributions

CZ and SW: conceptualization and supervision. LZ and JG: investigation and formal analysis, experiments, and analysis of results. CZ: funding acquisition. LZ, JG, and YS: writing original draft. CZ, YS, JG, and CZ: review and editing. All authors contributed to the article and approved the submitted version.

## Conflict of Interest

The authors declare that the research was conducted in the absence of any commercial or financial relationships that could be construed as a potential conflict of interest.
