# Supplementary material for: Acetic Acid-Producing Endophyte Lysinibacillus fusiformis Orchestrates Jasmonic Acid Signaling and Contributes to Repression of Cadmium Uptake in Tomato Plants
Source: Front Plant Sci. 2021 Jun 4;12:670216. doi: 10.3389/fpls.2021.670216 (PMC8211922; doi:10.3389/fpls.2021.670216)
Supplement: Supplementary file 1 [file Data_Sheet_1.ZIP › Supplementary_Material.docx]

Supplementary Material

**Figure 1.** Effects of soil drench with *L. fusiformis* Cr33 on the resistance of tomato plants to Cd stress. 10-d-old tomato plants were cultured in soil containing 0 or 100 mg Cd kg^-1^ soil with or without Cr33 for 4 weeks. These treated plants were used to examine shoot phenotypes (A), chlorophyll content (B), Fv/Fm (C), and shoot and root Cd content (D). Moreover, the population of Cr33 was quantified in the roots using qPCR analyses (E). Asterisks indicated significant differences between the control and inoculated plants (n = 8 biological replicates) using Student’s test (ns, not significant; *, *p* < 0.01; **, *p* < 0.01).

**Figure 2.** Root inoculation with *L. fusiformis* Cr33 increased root viability in tomato plants under Cd stress condition. Three-week-old tomato plants were cultured in the split-root systems containing 0 or 100 μM Cd with or without Cr33 for 10 and 20 d, respectively. These plants were used to measure root viability using TTC assays. Asterisks indicated significant differences between the control and inoculated plants (n = 8 biological replicates) using Student’s test (ns, not significant; *, *p* < 0.05; **, *p* < 0.01).

**Figure 3.** Effects of *L. fusiformis* Cr33 on rhizospheric organic acids released from tomato roots under Cd stress. 10-d-old tomato plants were cultured in soil containing 100 mg Cd kg^-1^ soil with or without Cr33 for 4 weeks. Root exudates from these treated plants were used to determine total organic acids (A), formic acid (B), acetic acid (C), lactic acid (D), oxalic acid (E), malic acid (F), succinic acid (G) and citric acid (H) by HPLC. Asterisks indicated significant differences between the control and inoculated plants (n = 8 biological replicates) using Student’s test (ns, not significant; *, *p* < 0.05; **, *p* < 0.01; ***, *p* < 0.0001).

**Figure 4.** [Qualitative](javascript:;) tests of acetic acid-producing bacteria. The culture of *L. fusiformis* Cr33 was neutralized with 0.1 M NaOH, and then reacted with 20 μL of 5% FeCl_3_, followed by heating for 10 min at 100°C. The color of the reaction mixture was detected for assessing the acetic acid-producing ability of Cr33.

**Figure 5.** Gene Ontology (GO) enrichment analyses for DEGs of both the Group I (Cd48 *vs* -Cd) and II (Cr33+Cd48 *vs* Cd48), including biological process, cellular component, and molecular function.

**Table 1.** Up- and down-regulated DEGs in the Group I (G1, +CONpcf vs –CONpcf).

**Table 2.** Up- and down-regulated DEGs in the Group II (G2, +CONpcf+Pst vs –CONpcf+Pst).

**Table 3.** Shared DEGs between up-regulated DEGs of the Group I (G1, +CONpcf vs –CONpcf) and the Group II (G2, +CONpcf+Pst vs –CONpcf+Pst).
