# Supplementary figures and images for: Acetic Acid-Producing Endophyte Lysinibacillus fusiformis Orchestrates Jasmonic Acid Signaling and Contributes to Repression of Cadmium Uptake in Tomato Plants
Source: Front Plant Sci. 2021 Jun 4;12:670216. doi: 10.3389/fpls.2021.670216 (PMC8211922; doi:10.3389/fpls.2021.670216)

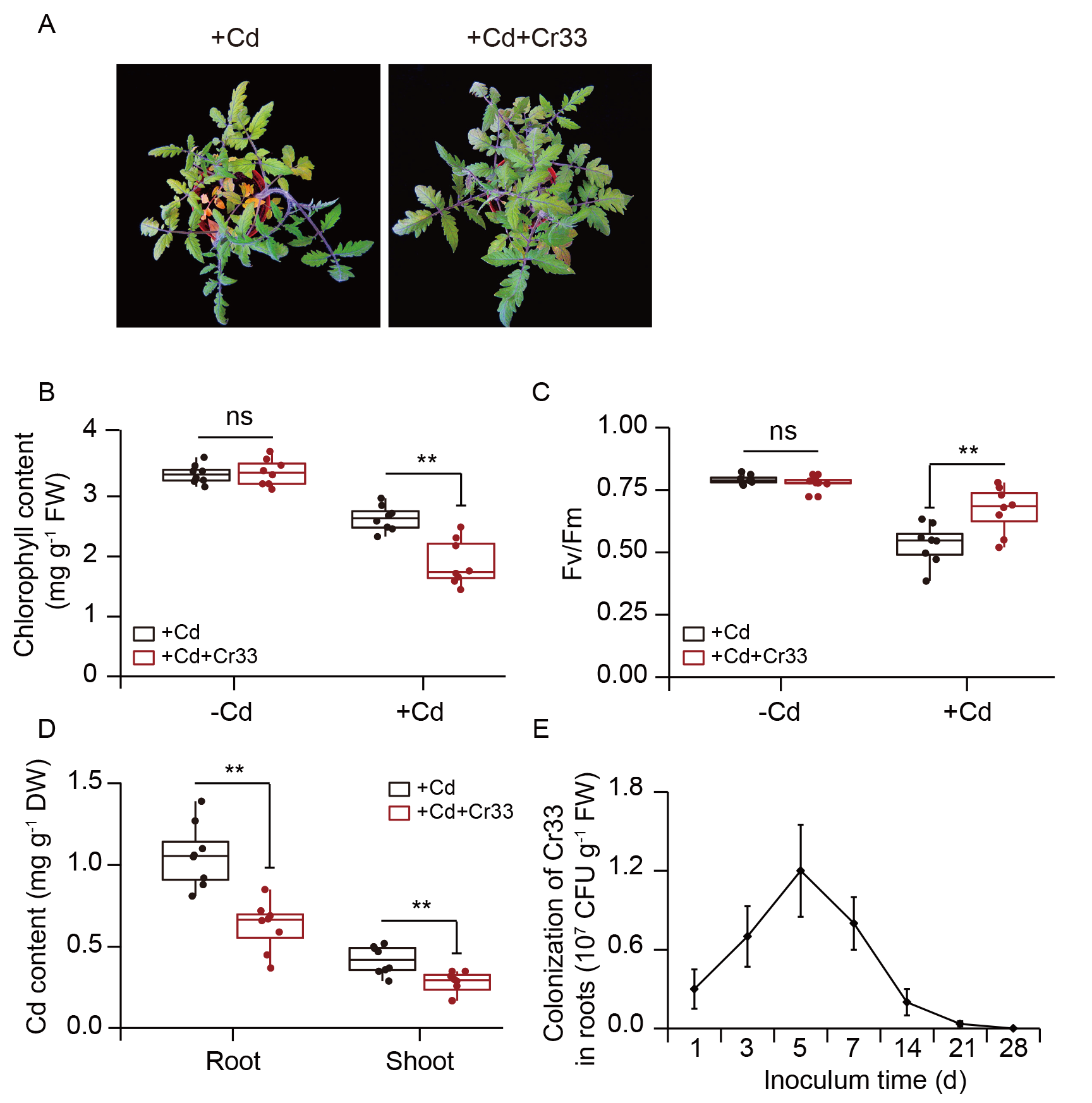

Supplement: Supplementary file 1 [file Data_Sheet_1.ZIP › Figure S1.tif]

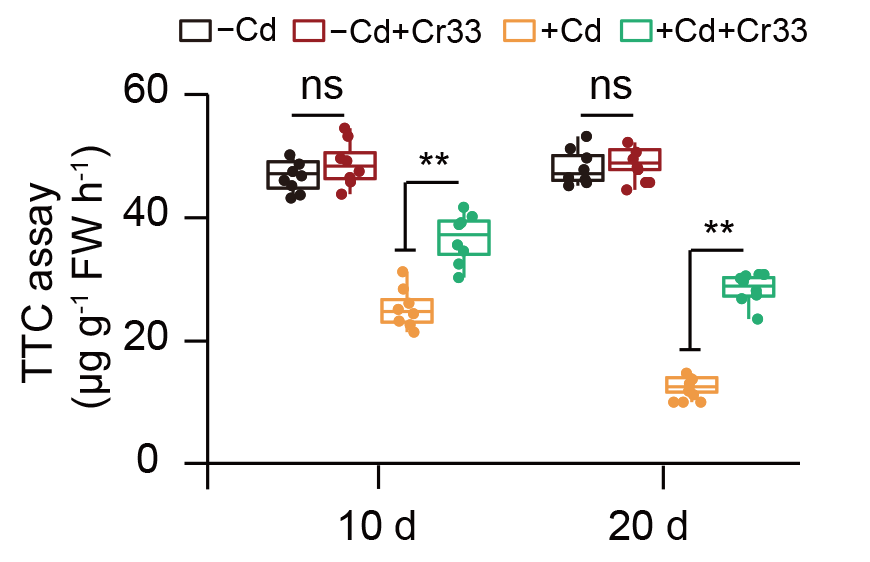

Supplement: Supplementary file 1 [file Data_Sheet_1.ZIP › Figure S2.tif]

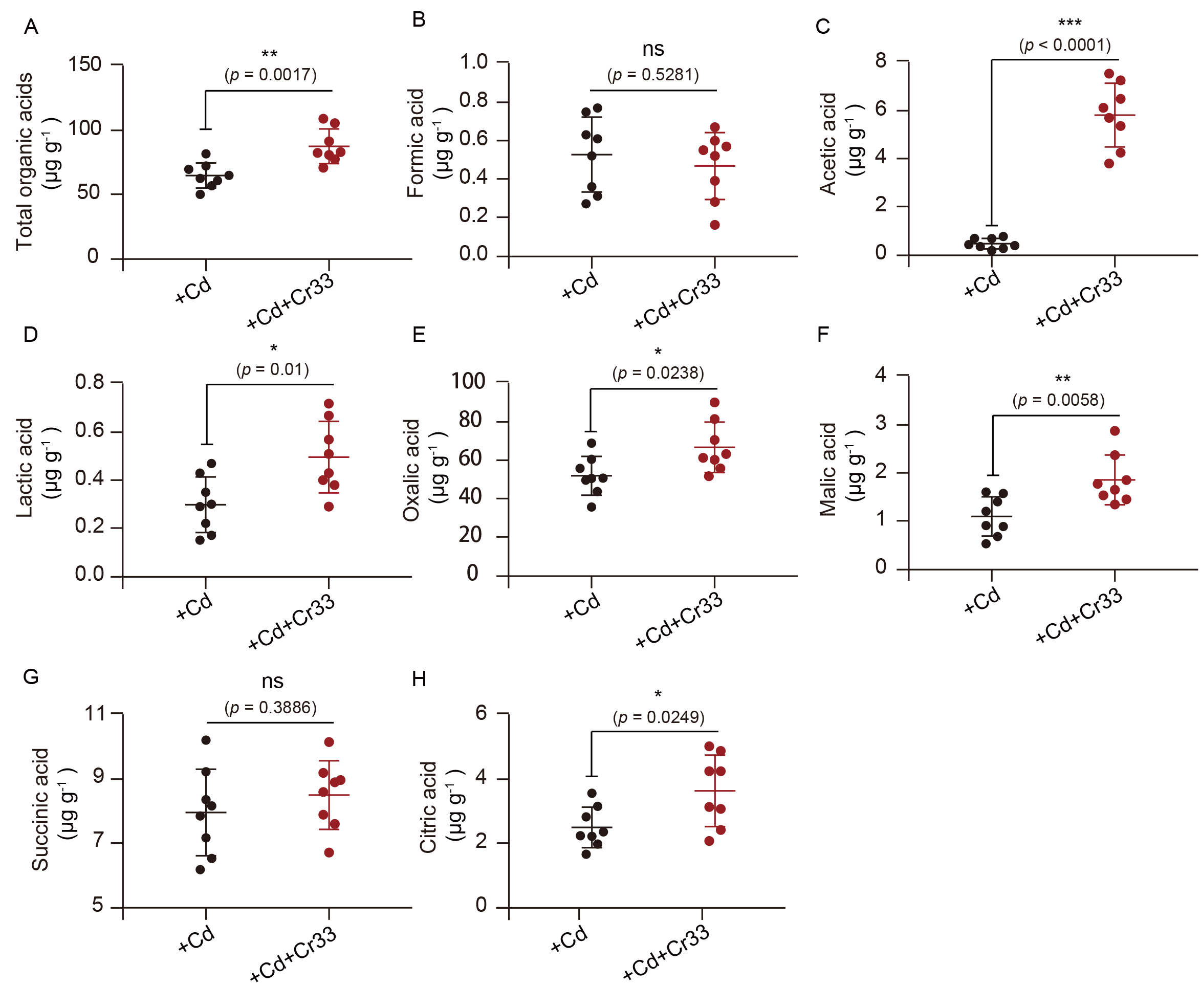

Supplement: Supplementary file 1 [file Data_Sheet_1.ZIP › Figure S3.tif]

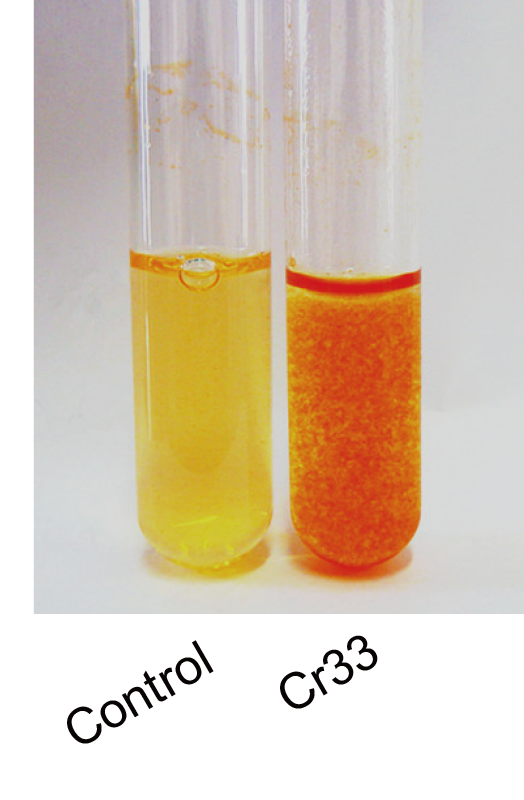

Supplement: Supplementary file 1 [file Data_Sheet_1.ZIP › Figure S4.tif]

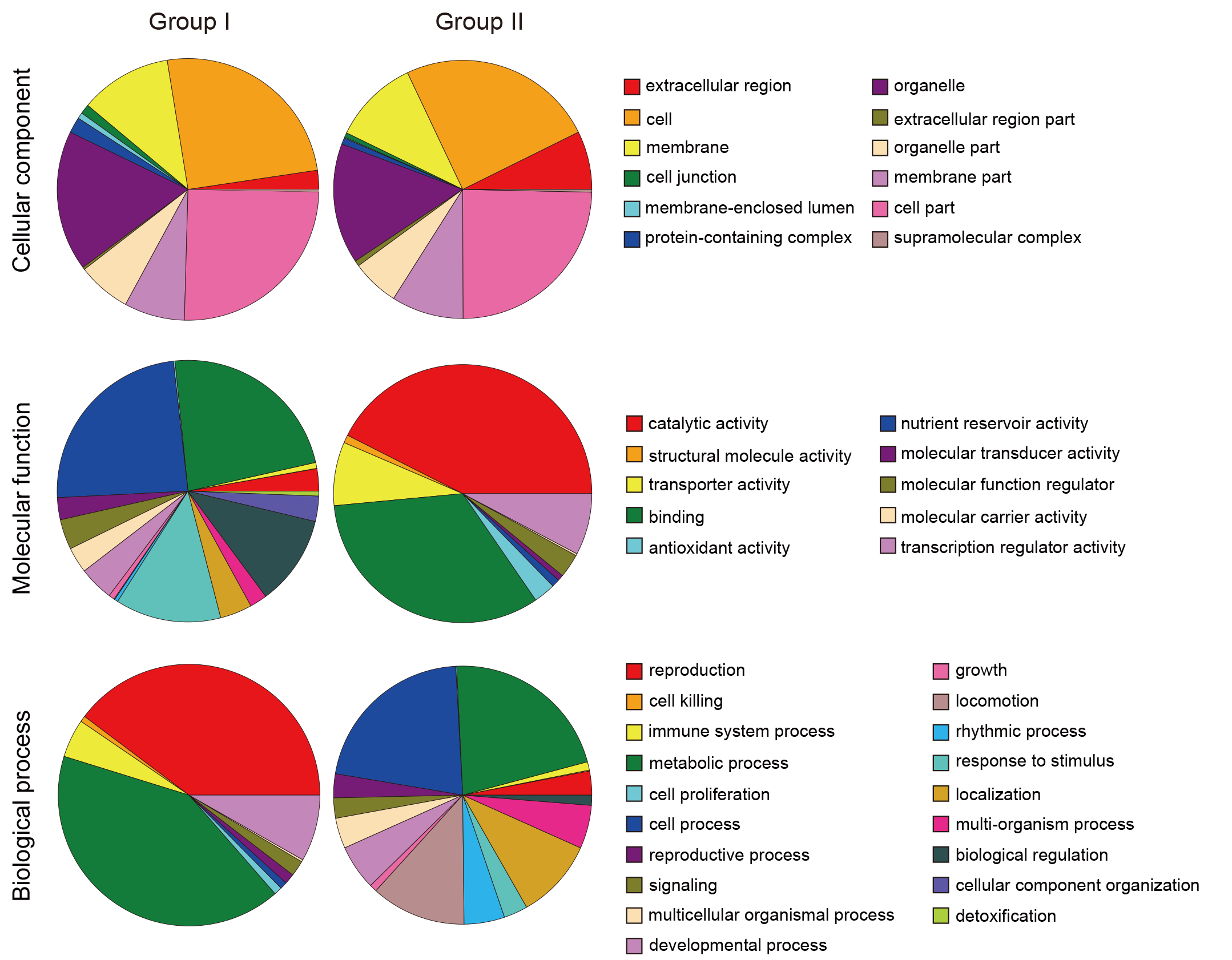

Supplement: Supplementary file 1 [file Data_Sheet_1.ZIP › Figure S5.tif]
